# Effects of zinc chloride–silicone oil treatment on wood dimensional stability, chemical components, thermal decomposition and its mechanism

**DOI:** 10.1038/s41598-018-38317-5

**Published:** 2019-02-07

**Authors:** Zhengbin He, Lijie Qu, Zhenyu Wang, Jing Qian, Songlin Yi

**Affiliations:** 1 Beijing Key Laboratory of Wood Science and Engineering, College of Material Science and Technology, Beijing Forestry University, No. 35, Qinghua East Road, Haidian District, Beijing 100083 P.R. China; 2 Faculty of Forestry, University of Toronto, 33 Willcocks Street, Toronto, Ontario M5S 3B3 Canada

## Abstract

The hygroexpansion and anisotropy of wood limit its application in construction and wood products industry. Zinc chloride–silicone oil was use to decrease the hygroscopicity and improve the dimensional stability of wood at 80 °C, 140 °C, 160 °C and 180 °C. The effects of the treatment on the dimensional stability, chemical structure, thermal degradation, morphology of wood were evaluated, and the mechanism was determined. Results indicated that the zinc chloride–silicone oil treatment at 80 °C improved the dimensional stability and decreased the hygroscopicity of wood. The tangential, radial, and volumetric swelling coefficients of the treated wood decreased by 9.7%, 33.5%, and 18.2%, respectively, relative to those of the untreated wood. Zinc chloride–silicone oil treatment also changed the chemical structure of wood by degrading the wood components and decreasing the moisture absorption groups. Moreover, zinc chloride–silicone oil treatment significantly influenced the thermal degradation of wood, as samples treated with zinc chloride–silicone oil at 140 °C, 160 °C and 180 °C presented sharp peaks around 511 °C, 501 °C and 473 °C. The control group exhibited a more common derivative thermogravimetric curve with a sharp peak at 375 °C. In addition, the silicone oil could impregnate wood, occlude moisture passage, and prevent the movement of moisture in wood. This method can be applied in building and wood industries to expand the applications of wood products.

## Introduction

Wood is a natural and renewable lignocellulosic material characterized by a high strength-to-weight ratio, low energy cost for processing, environmental sustainability^1–3^, natural degradation, and regulation of air temperature and humidity^4^. Wood has thus been used for construction, building, furniture, decoration^5,6^, and other applications in recent years. However, as a natural material containing cellulose, hemicellulose, and lignin, wood is also prone to hygroexpansion and anisotropy^7,8^, it shrinks and swells when equilibrium moisture content (EMC), which is controlled by environmental temperature and humidity, changes with the environmental conditions. Then, wood defects, such as cracks, transformation, and decay generated^9^, and markedly shortening the service life and value of wood products, thus limiting their wide application.

Thermal treatment of wood is an environmentally friendly method to improve the dimensional stability, weathering ability, and other characteristics of wood^10–13^. This method has been widely employed in various settings. However, while this approach is suitable for improving wood quality, it consumes substantial energy based on the scheduling requirement of high temperature and high investment cost; equipment must be heat-preserved and decay-resistant^14^. Therefore, a low-temperature heat treatment schedule should be applied to reduce energy costs.

Wood thermal treatment can potentially increase the dimensional stability of wood and other characteristics primarily because wood components undergo degradation under high-temperature conditions^15–17^. Zinc chloride solution is also weakly acidic and can be used as a catalyst to promote wood hemicellulose and cellulose degradation at reduced temperatures and decrease moisture absorption^18^. Therefore, zinc chloride was used as the catalyst, and silicone oil was used as the heating medium in the treatment of wood samples to determine the optimal schedule for zinc chloride–silicone oil treatment. The effects of zinc chloride–silicone oil treatment on the dimensional stability, chemical components, and thermal decomposition behavior of wood were evaluated to broaden the application of wood.

## Results and Discussion

### Dimensional stability of wood

**Figure 1:**
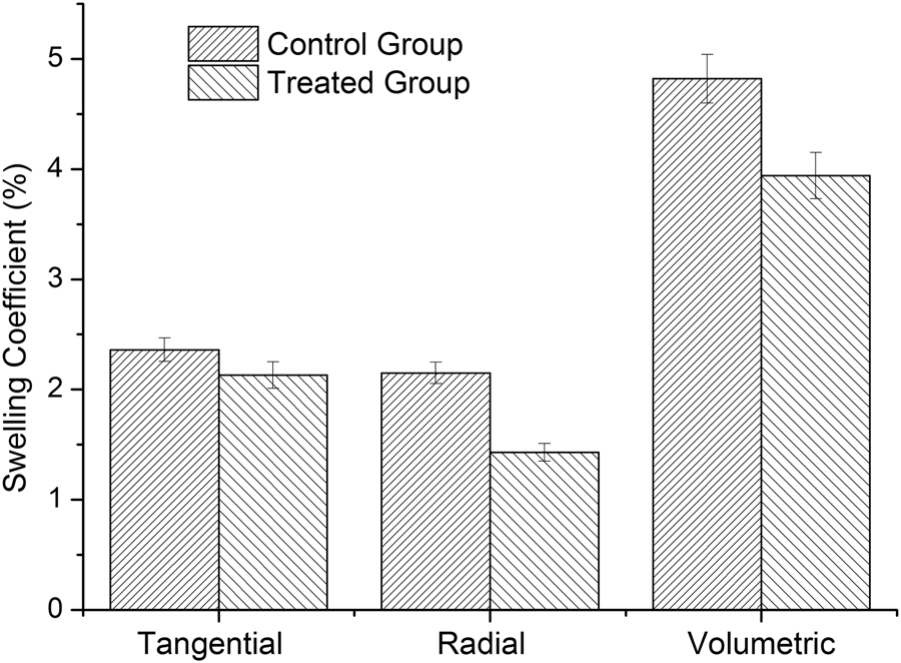
Effects of zinc chloride–silicone oil treatment on wood swelling.

The dimensional stability of wood is one of the most important parameters for wood applications. Wood with low dimensional stability can shrink or swell when the EMC in the surrounding environment decreases or increases^13^. Wood moisture also changes with variations in environmental temperature and humidity, leading to defects^19^. Therefore, the dimensional stability of wood significantly affects the quality and application of wood product. Moreover, the swelling coefficients in the tangential and radial directions are the most significant factors for estimating the dimensional stability of wood^7,13^. The samples were evaluated after treatment, and the results indicated that the samples treated at 140 °C, 160 °C and 180 °C were so degraded that its surface was carbonized, the sample failed to retain its original shape, and its structure was broken by the catalyst of zinc chloride at high temperature; meanwhile the samples treated at 80 °C in a silicone oil bath retain its intact structure. Figure 1 shows the effects of zinc chloride treatment in a silicone oil bath at 80 °C on the tangential swelling (TS), radial swelling (RS) and volumetric swelling (VS) coefficients of wood. TS coefficient varied from 2.36% to 2.13%, reflecting a decrease of 9.7%. RS coefficient decreased from 2.15% to 1.43%, reflecting a decrease of 33.5%. VS coefficient decreased from 4.82% to 3.94%, indicating a reduction of 18.2%. Thus, the zinc chloride-silicon oil treatment reduced the TS, RS, and VS coefficients of the wood, thus increasing its dimensional stability. This improvement in dimensional stability may be attributed to the following: (i) low acidity of the zinc chloride solution at a certain temperature, leading to hemicellulose degradation in wood^18,20–22^, and (ii) water repellency of the silicone oil, reducing the hygroscopicity of wood^6,23^.

### Fourier Transform Infrared (FTIR) Spectroscopy analysis

**Figure 2:**
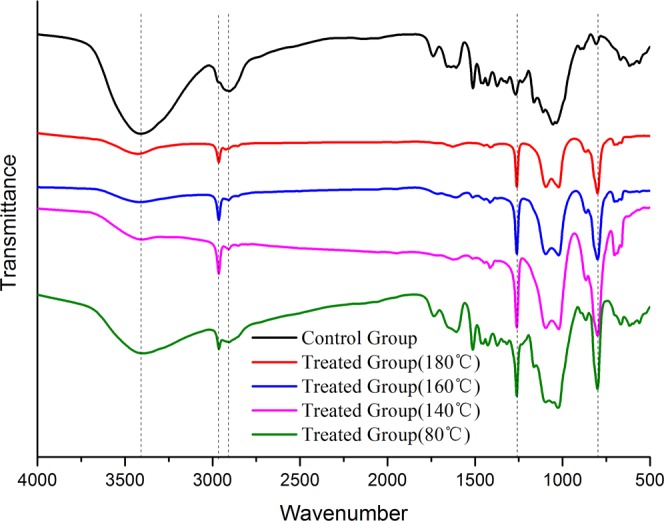
FTIR spectra of samples treated with zinc chloride in silicone oil bath.

The hygroscopicity of wood is directly influenced by the hygroscopic group, such as hydroxyl; variations in the chemical groups of wood can affect its dimensional stability^24–28^. FTIR spectroscopy is a suitable technique for measuring variations in chemical structure of treated samples^29–32^. The FTIR spectra of the samples treated with zinc chloride in silicone oil bath are presented in Fig. 2. Wood impregnated with zinc chloride and treated in silicone oil substantially influenced the chemical structures of the wood. The band intensity at 3423 cm^−1^ corresponding to -OH stretching^33^ and the band intensity at 2905 cm^−1^ corresponding to –CH stretching^34^ generally decreased relative to those of the control group. This result may be attributed to the degradation of hemicellulose and other components in the wood cell wall, which has free hydroxyl and can absorb moisture^35–38^. Thus, the dimensional stability of the wood increased^39,40^. Moreover, the band intensities at 2970 cm^−1^ corresponding to –CH_2_ stretching^41–43^ increased relative those of the control because of silicone oil impregnation. The samples impregnated with zinc chloride and treated in silicone oil exhibited markedly increased band intensities at 1263 cm^−1^ corresponding to Si-CH_3_ stretching in silicone oil. In addition, 800 cm^−1^ was assigned to the Si-C, Si-O, and Si-O-CH_3_ groups^44^, indicating that silicone oil was impregnated into wood. Therefore, hemicellulose and other hygroscopic groups in the cell wall of the wood impregnated with zinc chloride could be degraded, and water-repellent silicone oil was absorbed by wood, thereby reducing the hygroscopicity and improving dimensional stability of wood.

### Thermal decomposition by thermogravimetric (TG) and derivative thermogravimetric (DTG)

**Figure 3:**
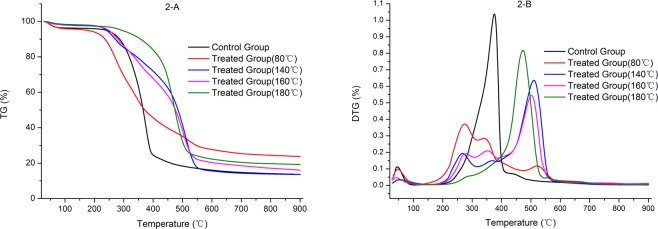
TG and DTG curves for the treated and untreated wood.

To evaluate the effects of zinc chloride–silicone oil treatment on the thermal characteristics of wood, the thermogravimetric (TG) and derivative thermogravimetric (DTG) curves of treated and untreated samples are presented in Fig. 3. Significant differences between the treated and untreated samples were observed. A small weight loss (approximately 1.1–3.7 wt%) was observed from the TG curve during the first stage (from 28 to 100 °C), which was the dehydration stage; no significant degradation of wood components was exhibited^29,30^. The weight loss decreased with an increase in treatment temperature because the wood immersed in the silicone oil and heated to a certain temperature underwent drying, and the rate of moisture loss increased with temperature. Residual moisture was also low in the treated samples. The temperature varied from 100 °C to 200 °C during the second stage under which some wood components might be degraded, and some complex structures could break their bonds^45–48^. The largest mass loss occurred during the third stage with temperatures ranging from 200 to 580 °C. During this stage, all structural components, including hemicellulose, cellulose, and lignin (66–81% of total mass) in the samples were thermally degraded^28,49^. Three peaks were observed for all samples. The samples impregnated with zinc chloride at 80 °C showed three peaks around 278 °C, 346 °C, and 525 °C; those treated at 140 °C showed three peaks at 278 °C, 354 °C, and 511 °C; those treated at 160 °C showed peaks at 278 °C, 355 °C, and 501 °C; and those treated at 180 °C showed peaks at 281 °C, 473 °C, and 557 °C. During the higher-temperature stage, the samples treated at 80 °C, 140 °C, 160 °C, and 180 °C showed peaks at 527 °C, 511 °C, 501 °C, and 473 °C, respectively, which might be the reason that more complex structures could break their bonds during silicone oil treatment. Consequently, samples are easily during the TG analysis. Moreover, the treated samples appeared different from the untreated samples. The control group showed a more common DTG curve with a sharp peak at 375 °C, which was attributed to cellulose, hemicellulose or lignin decomposition^50^. These differences could be attributed to zinc chloride being acidic in water, thus promoting wood degradation during treatment. Most wood components were degraded when the temperature increased to 180 °C^21^. During the fourth stage, the gradual mass loss was observed in the samples. The content of residual material varied between the control group and the treated group, which could be attributed to the incomplete degradation of the impregnated zinc chloride and silicone oil during thermal degradation.

### Morphology and mechanism

#### Morphological analysis by Scanning Electron Microscopy (SEM)

**Figure 4:**
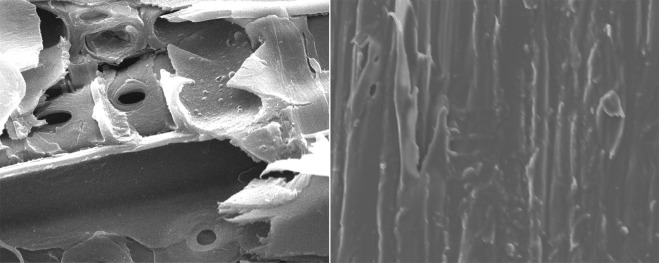
SEM micrographs (800×) for the control group (left) and the group treated at 80 °C (right).

Figure 4 presents the morphology of the untreated (left) and treated (right) samples. The samples impregnated with zinc chloride and treated at 80 °C significantly influenced the morphology of the wood. Most porosity (e.g., pits) of the treated samples was occluded, and the internal surface of the wood was smoother than that of the control group. Some components were degraded during treatment process, thereby occluding the internal water passage in wood and decreasing numbers of the hygroscopic group. These features improve the dimensional ability of wood, reduce its hygroscopicity, and expand its applications in buildings and furniture.

#### Mechanism

**Figure 5:**
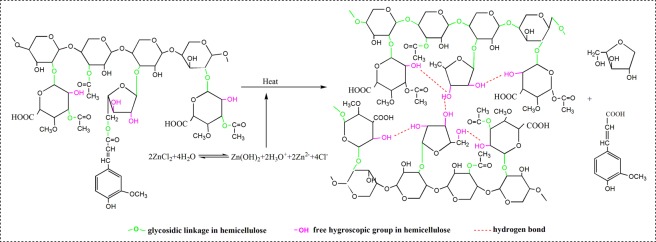
Mechanism of wood degradation during treatment.

On the basis of the aforementioned results, wood impregnated with zinc chloride and treated in silicone oil exhibits increased dimensional stability, reduced hydroxy content, and lowered hygroscopicity. Zinc chloride is hydrolyzed in water and generates acid and zinc ion. The impregnation of wood with solution might have increased the degradation of moisture absorption groups. A tentative mechanism is presented in Fig. 5. Hemicellulose, one of the components in wood, contains moisture-absorbing groups (i.e., hydroxy), and most of the hydroxyl on its branched chain can absorb or lose moisture, along with variations in temperature and humidity^35–37^. These factors can generate defects, affect the quality of wood, and limit its applications. The branched chain in the hemicellulose is degraded in a weak acid environment at certain temperatures, with zinc chloride as the catalyst^20,21^. Consequently, some branched chain degraded from the hemicellulose is dissolved in the solution; simultaneously, the distances between some hydroxyl on the hemicellulose branched chain is close, and the hydrogen bond was established. Thus the free hydroxyl, which could absorb the moisture in the hemicellulose, decreased, thereby reducing the amount of water that could be absorbed by wood^18,22^. Thus, a decrease in hygroscopic group improved the wood dimensional stability and reduced the hygroscopicity of wood during application.

## Conclusions

The zinc chloride–silicone oil treatment can improve the dimensional stability and decrease the hygroscopicity of wood. The method can also reduce the swelling coefficient of wood at 80 °C. The TS, RS and VS coefficients of the treated wood decreased by 9.7%, 33.5%, and 18.2%, respectively, relative to those of the control group. The treatment conducted at certain temperatures also affected the chemical structure, degraded the hemicellulose, and decreased the hydroxyl content and other components of wood. Moreover, the zinc chloride–silicone oil treatment largely influenced the thermal degradation of wood. The thermal degradation generally includes four stages, revealing significant differences between the treated and untreated samples during the third stage (200–580 °C), with approximately 66–81% of total mass degraded. Three peaks were observed for all samples. At high temperature stages, the peaks of the samples treated at 80 °C, 140 °C, 160 °C and 180 °C were observed around 527 °C, 511 °C, 501 °C and 473 °C; meanwhile, the control group demonstrated a more commonly observed DTG curve with a sharp peak at 375 °C. In addition, the samples impregnated with zinc chloride and treated at 80 °C significantly influenced the wood morphology. Thus, silicone oil impregnated the wood and occluded water passage (e.g., pits). Several internal components of wood could thus degrade and potentially decrease the hygroscopicity and increase the dimensional stability of wood. The zinc chloride–silicone oil treatment can improve the dimensional stability and decrease the hygroscopicity of wood because zinc chloride generates weak acids and the catalyst of zinc chloride can promote hemicellulose degradation and decrease moisture absorption in wood. In addition, silicone oil can occlude water passage and prevent the migration of moisture into the internal wood.

## Materials and Methods

### Materials

Chinese fir (*Cunninghamia lanceolata (Lamb*.*) Hook*), a type of fast-growing wood planted across large areas of China, was taken as the experimental material. It was obtained from Sichuan Province, China. The dimension of the test specimens was 20 mm (tangential) × 20 mm (radial) × 20 mm (longitudinal) with an initial moisture content of (80 ± 5) % according to GB/T 1931–2009 standard^51^.

### Wood heat treatment

#### Impregnation with zinc chloride

In this study, 120 wood samples with a moisture content of 80% were immersed in a zinc chloride solution at 2% w/w and then treated in a vacuum chamber at 0.005 MPa for 1.0 h. Pressure was then recovered to atmospheric pressure, and vacuuming and the reverse process were performed thrice to impregnate the samples with zinc chloride.

#### Heat treatment

After impregnation with zinc chloride, the 120 wood samples were divided into four groups, each of which was immersed into a silicone oil bath at room temperature and then treated at 80 °C, 140 °C, 160 °C, and 180 °C with a heating rate of 15 °C/min. The samples were then treated for 2 h when the temperature of the silicone oil reached target temperature.

### Dimensional stability of wood

A total of 20 wood samples were used to measure the dimensional stability of wood, and swelling tests were performed in accordance with the GB/T 1931–2009 standard^48^. The treated and control groups were oven-dried and subsequently stored in a climate-controlled chamber at 20 °C and 65% humidity to reach the EMC. The dimensions were measured before and after conditioning. The swelling coefficients were determined using Eq. (1):

1 $$a=\frac{{l}_{w}-{l}_{0}}{{l}_{0}}\times 100 \% $$

where *a* is the swelling coefficient (radial, tangential, and volumetric), (%); *l*_0_ denotes the initial dimension of the samples (mm); and *l*_*w*_ represents the dimension after conditioning (mm).

### Chemical structure analysis

FTIR was used to analyze the chemical structure of the wood with the use of a standard FTIR spectroscope (Tensor 27; Bruker, Germany). A total of 32 scans were conducted for each sample, recorded in 4000–500 cm^−1^ range at a resolution of 2 cm^−1^ in the transmission mode.

### Thermogravimetric analysis (TGA)

TGA was used to compare the degradation characteristics of the treated and untreated samples. The thermal stability of each sample (6.56 mg) was determined using a thermogravimetric analyzer (Netzsch STA449F3; Germany) at a heating rate of 10 °C/min and final temperature of 900 °C in a nitrogen environment with a flow rate of 60 mL/min.

### Morphological characteristics

The surface shape of the samples was assessed based on morphological by SEM (Hitachi S-3400N II; Tokyo, Japan) to investigate potential variations in physical structure.
